# SCAU-Net: Spatial-Channel Attention U-Net for Gland Segmentation

**DOI:** 10.3389/fbioe.2020.00670

**Published:** 2020-07-03

**Authors:** Peng Zhao, Jindi Zhang, Weijia Fang, Shuiguang Deng

**Affiliations:** ^1^First Affiliated Hospital, Zhejiang University School of Medicine, Hangzhou, China; ^2^College of Computer Science and Technology, Zhejiang University, Hangzhou, China

**Keywords:** deep learning, semantic segmentation, attention mechanism, medical image, gland

## Abstract

With the development of medical technology, image semantic segmentation is of great significance for morphological analysis, quantification, and diagnosis of human tissues. However, manual detection and segmentation is a time-consuming task. Especially for biomedical image, only experts are able to identify tissues and mark their contours. In recent years, the development of deep learning has greatly improved the accuracy of computer automatic segmentation. This paper proposes a deep learning image semantic segmentation network named Spatial-Channel Attention U-Net (SCAU-Net) based on current research status of medical image. SCAU-Net has an encoder-decoder-style symmetrical structure integrated with spatial and channel attention as plug-and-play modules. The main idea is to enhance local related features and restrain irrelevant features at the spatial and channel levels. Experiments on the gland dataset GlaS and CRAG show that the proposed SCAU-Net model is superior to the classic U-Net model in image segmentation task, with 1% improvement on Dice score and 1.5% improvement on Jaccard score.

## 1. Introduction

In clinical practice, biomedical image analysis (Litjens et al., 2017) provides doctors with digital and quantitative medical information, and helps doctors make objective and accurate diagnosis. Image segmentation is a basic problem in medical image analysis. In short, it is to identify the target area in an image and distinguish the research object from the background. For instance, glands are important tissues of the human body that secrete special proteins and hormones. Malignant tumors caused by glandular differentiation, i.e., adenocarcinoma, is a common form of cancer. Different grades of differentiated glands have various morphological structures. In pathological examination, pathologists usually use Hematoxylin and Eosin (H&E) to stain glandular tissues, then evaluate the malignancy of adenocarcinoma and determine the grade of cancer (Niazi et al., 2019). Early detection of glandular differentiation can greatly improve the cure rate of patients, and these treatment methods often require detailed gland information, such as the size, shape and location of the glands before and after treatment, in order to propose a suitable treatment plan. At present, this work is mainly performed by expert pathologists. However, the morphology of glands in different histological differentiation grads is quite complex, and the texture and size vary from patient to patient. It is still a very challenging task.

Manually detecting and segmenting medical images consumes a lot of energy and time of doctors. In recent years, with the deepening cooperation between artificial intelligence and medical image analysis, the research of computer-aided medical image segmentation have exploded. Computer automatic segmentation enables doctors to quickly and easily obtain image markers related to the disease treatment process, detect malignant tumors early in time. Especially for the automatic segmentation of H&E gland images, pathologists can quickly extract important morphological features from massive histological images. This work helps pathologists to provide services to more patients while ensuring diagnostic accuracy. To some extent, it can solve the problem of imbalanced distribution of medical resources and lack of expert pathologists.

In this paper, we propose a deep learning network named Spatial-Channel Attention U-Net (SCAU-Net) for gland segmentation. The contributions of this paper are as follows:

1. Our model has a symmetrical structure. It exploits skip connections to concatenate outputs of encoder to the decoder in corresponding level. Multi-level features are fused to improve segmentation results.
2. We introduce spatial attention and channel attention as plug-and-play modules for the basic encoder-decoder structure. The module exploits hidden layer neural network to capture the non-linear relationship between spatial-wise and channel-wise feature, and essentially introduces a self-attention mechanism. The attention module performs feature recalibration to enhance local related features and restrain irrelevant features at the spatial and channel levels.

## 2. Related Work

### 2.1. Biomedical Image Segmentation

Computer automatic image segmentation algorithms are categorized as traditional algorithms based on manual features and deep learning algorithms based on Convolutional Neural Networks (CNNs) (Krizhevsky et al., 2012).

The main idea of traditional image segmentation algorithms is to segment the image into regions with similar properties, such as color and texture (Sharma and Aggarwal, 2010). Divided in principle, including the following types of methods: (1) Edge based segmentation. Algorithm exploits discontinuity principle such as grayscale and color to detect boundaries between regions (Hancock and Kittler, 1990; Liow, 1991). Fuzzy boundaries and noise can easily affect the performance of the method. (2) Region based segmentation. Pixels with similar properties are aggregated to form a complete object regions. Wu et al. (2005) proposed a intestinal gland images segmentation based on iterative region growing. The segmentation results of this method are sensitive to the number of clusters and regions initialization. (3) Textural feature based segmentation. This method divides the image regions according to texture properties (Sirinukunwattana et al., 2015).

In recent years, deep learning has become the main research method in many fields, and CNN is widely used in many different computer vision tasks. Unlike previous traditional methods, CNN is a data-driven method that can automatically learn advanced features from image without the need for artificial feature design and prior knowledge. In the medical field, CNN has also achieved good results in the detection and segmentation of cells (Raza et al., 2017), pancreas (Roth et al., 2015), liver tumors (Dou et al., 2016; Christ et al., 2017), glands (Chen et al., 2016; Xu et al., 2016; Yang et al., 2017; Graham et al., 2019), and other human tissues.

The full convolutional network (FCN) (Long et al., 2015) is the first method for image semantic segmentation using end-to-end deep neural networks. The innovation is that the fully connected layer is replaced by fully convolutional layer. This important innovation enables the network to adapt to the input of any resolution.

Datasets containing large amounts of labeled images have been established in other fields, such as ImageNet, COCO, etc. However, in the field of medical images, due to the high annotation cost, it is almost impossible to provide such a large dataset. Therefore, how to train a good model in the case of small datasets is a difficult research point. U-Net (Ronneberger et al., 2015) is based on the FCN structure, and exploits skip connections to transfer and fuse the output of feature maps with different resolutions to obtain more accurate outputs. It is firstly used for segmentation of neuron and cell images and has excellent performance on many medical image datasets. In the last few years of medical image segmentation, many works have been developed and improved on the basis of the U-Net (Çiçek et al., 2016; Milletari et al., 2016; Gordienko et al., 2018; Zhou et al., 2018). Unlike many recent studies focus on instance segmentation (Xu et al., 2016; Graham et al., 2019; Yu et al., 2020), SCAU-Net proposed in this paper extends U-Net as basic model in order to improve the accuracy of segmentation while retaining the original advantages. In addition, our method can be easily extended to other medical image segmentation such as liver, cell, etc.

### 2.2. Vision Attention

When looking at a scene, we often firstly scan the whole scene quickly and focus on the region of interest (ROI). This selective attention mechanism that mimics the Human Visual System (HVS) has been widely used in computer vision (Itti and Koch, 2001; Wang and Shen, 2017). There is no strict mathematical definition of the attention mechanism. Oktay et al. (2018) proposed a network of encoder-decoder-style called Attention U-Net, which exploits a Attention Gates control. Another modular attention mechanism is called self-attention. The computation and parameter overhead of the feature map's attention generation process is much smaller, which can be used as a plug-and-play module of the existing basic CNN architecture. This method introduces additional neural network modules, which can assign different weights to spatial-wise or channel-wise.

Spatial attention learns to focus on spatial location (where), and weights are assigned to each pixel. Therefore, the form of weights is a *H*×*W* 2D matrix. Jaderberg et al. (2015) introduced a learnable Spatial Transformer module, which can learn the location of object regions by the input feature map.

Channel attention learns to select important feature dimensions (what), and weights are assigned to each channel. Therefore, the form of weights is a 1D vector. Hu et al. (2018) proposed the Squeeze-and-excitation (SE) module, which learns the non-linear relationship between channels and performs dynamic channel-wise feature recalibration.

In addition, spatial and channel attention modules can be combined in a parallel or sequential manner. e.g., Dual Attention Network (Fu et al., 2019) parallels spatial and channel attention and fuses output features of attention module. Woo et al. (2018) proposed Convolutional Block Attention Module (CBAM), which sequentially builds the channel and spatial attention modules. Non-Local attention (Wang et al., 2018) computes the response at a position by capturing long-range dependencies at all positions. Bottleneck attention module (Park et al., 2018) generates a 3D attention map in two streams, i.e., spatial stream and channel stream.

## 3. Method

**Figure 1:**
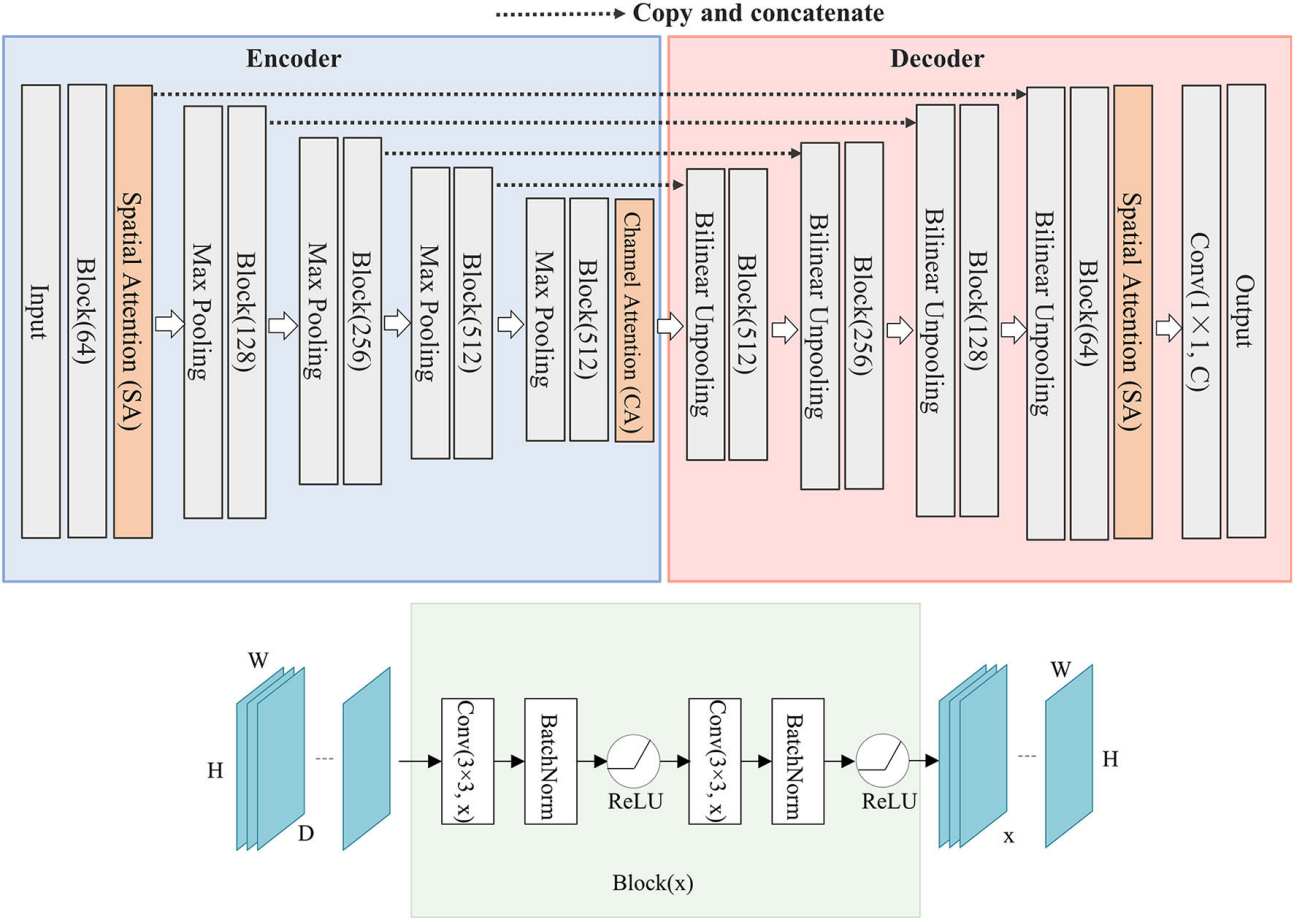
The structure of SCAU-Net. The entire structure is divided into four parts: encoder, decoder, spatial attention module, channel attention module. Given an input feature map of size D×H×W, the output size of Block(x) is x×H×W. W, width; H, height of feature map; D, input channel number; x, output channel number.

Inspired by U-Net network structure and attention mechanism, we propose a deep learning network named SCAU-Net. The entire structure is shown in Figure 1.

We define “Block(x)” which executes a 3×3 convolution followed by a batch normalization and ReLU activation, two times. x refers to the output channel number. The role of the encoder part is to extract features from the image and obtain compressed expression of the image features at multi-level. Down-sampling is performed by 2×2 max-pooling operation. During each down-sampling, the image size is reduced and the number of feature channels is doubled. The role of the decoder part is to gradually restore the details and spatial dimensions of the image according to the image features, and obtain the result of image segmentation mask. Up-sampling is performed by bilinear interpolation. Finally, a 1×1 convolutional layer is applied to predict the class of each pixel, denoted as Conv(1×1, C), where C is the number of classes. For image semantic segmentation, C is set to 2. The decoder part has a symmetrical structure to the encoder part. The copy operation links the corresponding down-sampling and up-sampling feature maps. The feature map is a combination of high-level and low-level features, and multi-level features are fused.

The medical image structure is simpler and more fixed than other images. For gland slices, the shooting angle and position are fixed, and the glands of approximate differentiation degree are often similar in shape. Inspired by the work of SE (Hu et al., 2018) and CBAM (Woo et al., 2018), we propose spatial attention module and channel attention module, which are used as plug-and-play modules in the network. Attention will focus on the objects and ignore the cluttered background. Especially, model will pay more attention on the edges of the glands because the fuzzy edge is the most worthy of the segmentation task.

### 3.1. Spatial Attention

**Figure 2:**
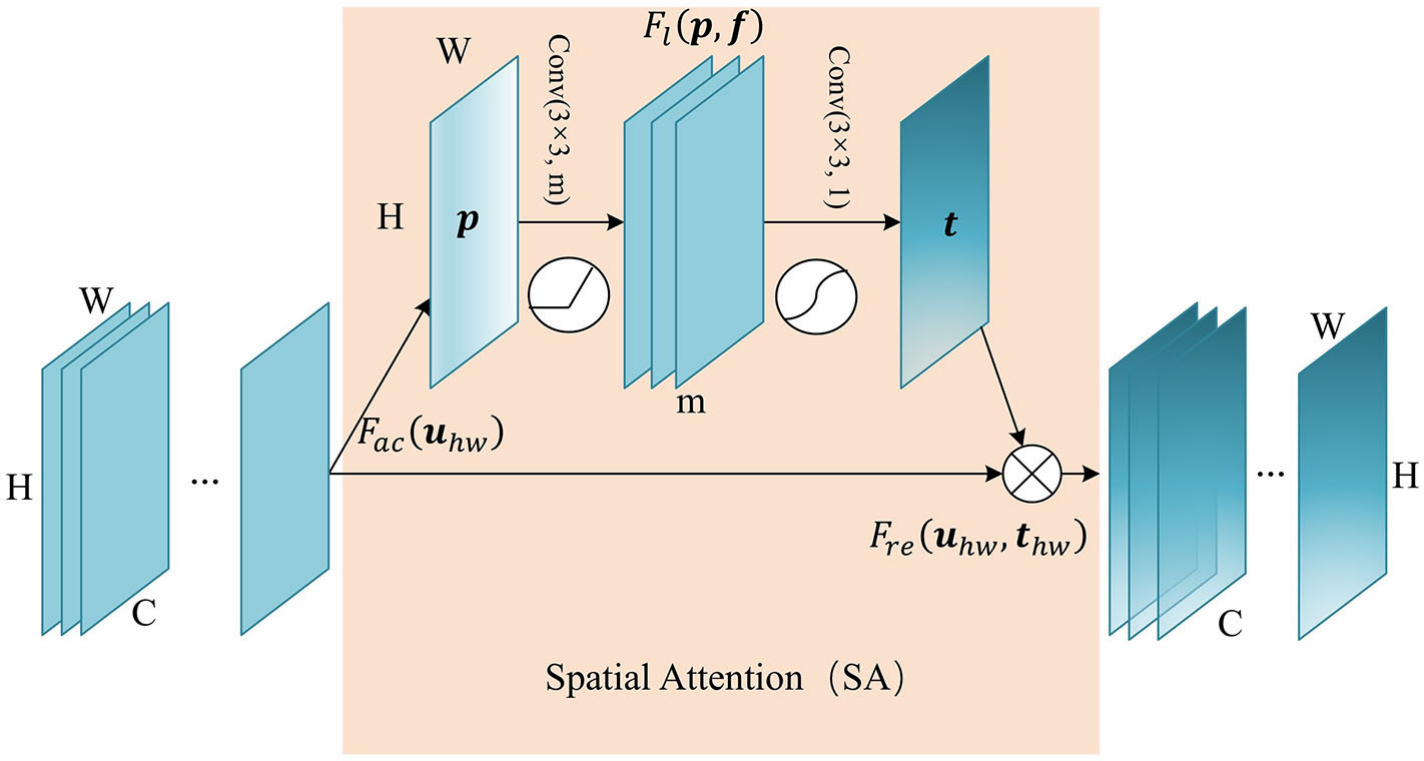
The structure of Spatial Attention (SA) module. The aggregate function *F*_*ac*_ generates a spatial descriptor ***p*** ∈ ℝ^*H*×*W*^. Self-learning function *F*_*l*_ implemented by two convolutional layers generates the spatial weights map ***t*** ∈ ℝ^*H*×*W*^. Finally, function *F*_*re*_ uses ***t*** to generate the output of the SA module.

Attention in the spatial-wise ignores the information of the channel, and treats the features of different channels equally. We add the spatial attention module to the low-level feature map since the low-level feature map mainly extracts the spatial feature such as contour, edge, with fewer channels. The module self-learns the interaction of spatial points, enhance key areas, and restrain irrelevant areas. The structure of the spatial attention module is shown in Figure 2. Firstly we pass the feature map ***U*** ∈ ℝ^*C*×*H*×*W*^ to the aggregation operation, which generates a spatial descriptor ***p*** ∈ ℝ^*H*×*W*^ by aggregating the feature map in its channel dimension (*C*). It generates a global distribution of spatial features:

(1) $$\begin{array}{r} p_{hw}=F_{ac}\left(u_{hw}\right)=\frac{1}{C}\overset{C}{\underset{i=1}{\sum }}u_{hw}\left(i\right) \end{array}$$

where $u_{hw}\in {\mathbb{R}}^{C}$ refers to the local feature at spatial position (*h, w*). The aggregate function *F*_*ac*_ uses global average pooling for channel dimension.

This is followed by a weight self-learning operation. It is implemented by convolutional layers. The function *F*_*l*_(***p***, ***f***) aims to fully capture the spatial correlation and adaptively generates the spatial weights map ***t*** ∈ ℝ^*H*×*W*^. The calculation formula is as follows:

(2) $$\begin{array}{r} t=F_{l}\left(p,f\right)=\sigma \left(g\left(p,f\right)\right)=\sigma \left(f_{2}\delta \left(f_{1}p\right)\right) \end{array}$$

where ***f*_1_** refers to 3×3 convolution, denoted as Conv(3×3, m), and ***f*_2_** refers to 3×3 convolution, denoted as Conv(3×3, 1). m refers to the channel number of hidden feature map. δ refers to activation function ReLU, and σ is a sigmoid activation function used to generate spatial weight ***t***_*hw*_ ∈ (0, 1), at position (*h, w*). In essence, the convolution operation that takes the original spatial descriptor as input can be considered as a spatial-wise self-attention function, and it can capture the non-linear inter-spatial relationship.

The weights calculated in the previous step are applied to the feature map ***U***. By spatial-wise recalibration *F*_*re*_(***u***_*hw*_, ***t***_*hw*_), the feature values of different position in ***U*** are multiplied by different weights to generate the output ***U***′ of the SA module:

(3) $$\begin{array}{r} u_{hw}^{\prime }=F_{re}\left(u_{hw},t_{hw}\right)=u_{hw}\cdot t_{hw} \end{array}$$

### 3.2. Channel Attention

**Figure 3:**
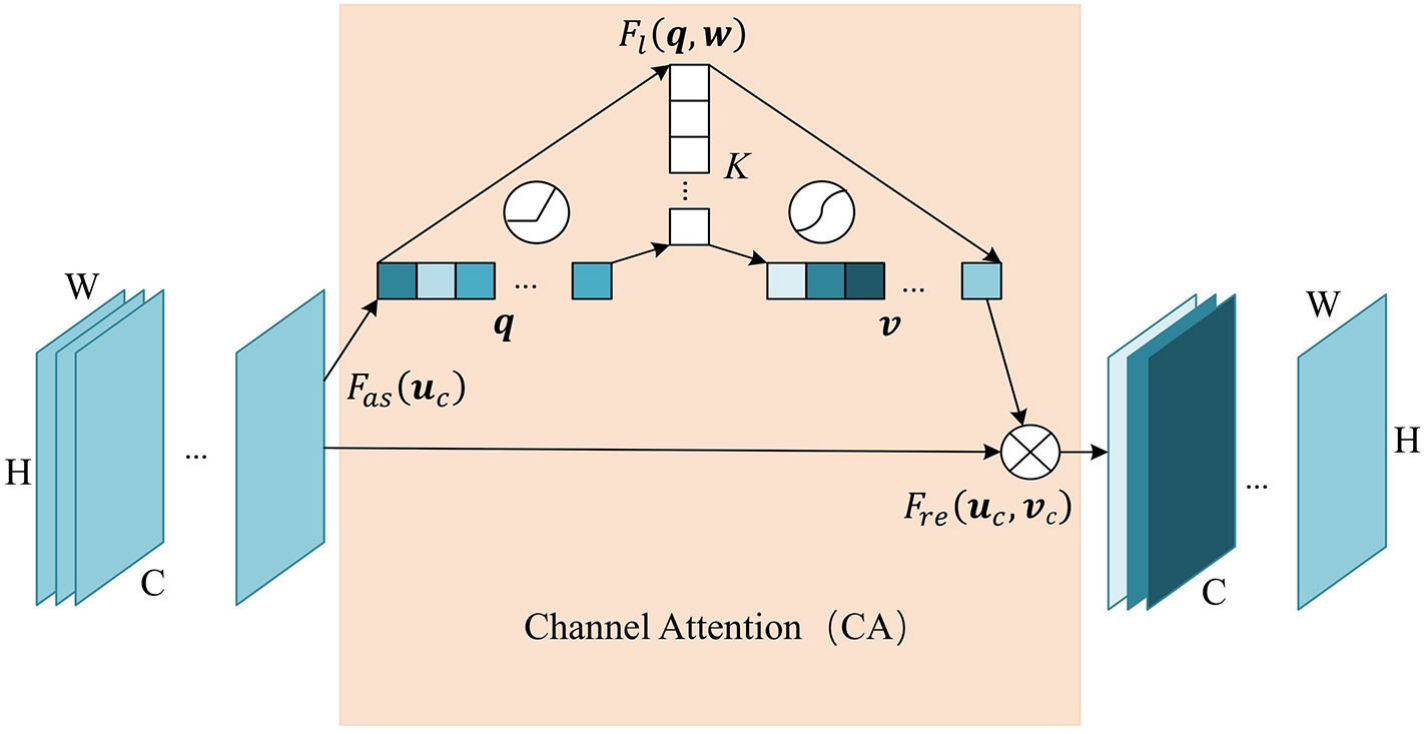
The structure of Channel Attention (CA) module. The aggregate function *F*_*as*_ generates a channel descriptor ***q*** ∈ ℝ^*C*^. Self-learning function *F*_*l*_ implemented by two fully connected layers generates the channel weights map ***v*** ∈ ℝ^*C*^. Finally, function *F*_*re*_ uses ***v*** to generate the output of the CA module.

Similarly, we add the channel attention module at the last layer of the encoder, since the hight-level feature map mainly expresses complex feature with large receptive field and more channels. This mechanism allows the network to perform feature recalibration, through learning to exploit global information to selectively enhance useful features and restrain useless features. The structure of the channel attention module is shown in Figure 3. Firstly we pass the feature map ***U*** ∈ ℝ^*C*×*H*×*W*^ to the aggregation operation, which generates a channel descriptor ***q*** ∈ ℝ^*C*^ by aggregating the feature map in its spatial dimension (*H*×*W*). It generates a global distribution of channel features:

(4) $$\begin{array}{r} q_{c}=F_{as}\left(u_{c}\right)=\frac{1}{H\times W}\overset{H}{\underset{i=1}{\sum }}\overset{W}{\underset{i=j}{\sum }}u_{c}\left(i,j\right) \end{array}$$

where $u_{c}\in {\mathbb{R}}^{H\times W}$ refers to the local feature of channel *c*. The aggregate function *F*_*as*_ uses global average pooling for spatial dimension.

This is followed by a weight self-learning operation. It is implemented by fully connected layers. The function *F*_*l*_(***q***, ***w***) aims to fully capture the dependencies between channels and adaptively generates the channel weights map ***v*** ∈ ℝ^*C*^. The calculation formula is as follows:

(5) $$\begin{array}{r} v=F_{l}\left(q,w\right)=\sigma \left(g\left(q,w\right)\right)=\sigma \left(w_{2}\delta \left(w_{1}q\right)\right) \end{array}$$

where $w_{1}\in {\mathbb{R}}^{K\times C}$, $w_{2}\in {\mathbb{R}}^{C\times K}$. *K* refers to number of hidden neurons. σ is a sigmoid activation function used to generate channel weights ***v***_*c*_ ∈ (0, 1), at channel *c*. With fully-connected hidden layers, it can capture the non-linear interaction between channels.

The weight calculated in the previous step is applied to the feature map ***U***. By channel-wise recalibration *F*_*re*_(***u***_*c*_, ***v***_*c*_), the feature values of different channels in ***U*** are multiplied by different weights to generate the output ***U***′ of the CA module:

(6) $$\begin{array}{r} u_{c}^{\prime }=F_{re}\left(u_{c},v_{c}\right)=u_{c}\cdot v_{c} \end{array}$$

## 4. Experiments and Results

### 4.1. Dataset

The two gland datasets used in the experiments are provided by a team of pathologists at the University Hospitals Coventry and Warwickshire, UK. (1) Gland Segmentation Challenge Contest (GlaS) (Sirinukunwattana et al., 2015) in MICCAI 2015. (2) The colorectal adenocarcinoma gland (CRAG) (Graham et al., 2019) dataset. The images are Haematoxylin and Eosin (H&E) stained slides of a variety of histologic grades. The GlaS dataset is split into 85 training images (benign/malignant = 37/48) and 80 testing images (benign/malignant = 37/43). We random split from 165 images using 80% images as the training set and the remaining 20% for testing. Images are mostly of size 780×520 pixels. The CRAG dataset is split into 173 training images and 40 test images. Images are mostly of size 1,510×1,510 pixels. And the ground truth annotations of the glands are provided by expert pathologists.

All the images processed by the network have fixed size of 512×512 pixels. Since the dataset is small, the training data is extended by using the data augmentation method in our experiments, i.e., a series of random changes such as rotation, scaling, cropping, etc., to increase the robustness and reduce overfitting.

### 4.2. Experimental Setting

The proposed network was implemented using Pytorch (Paszke et al., 2019) deep learning framework. Experiments are carried out on Ubuntu 16.04 operating system, NVIDIA Tesla K80 GPU, CUDA 10.1.

### 4.3. Training Process

The loss function defined in experiment is a combination of cross-entropy loss and dice loss:

(7) $$\begin{array}{r} CELoss=-\frac{1}{n}\sum y*log\left(y^{\prime }\right)+\left(1-y\right)*log\left(1-y^{\prime }\right) \end{array}$$

(8) $$\begin{array}{r} DiceLoss=\frac{2\sum \left(y^{\prime }*y\right)}{\sum y^{\prime }+\sum y} \end{array}$$

(9) $$\begin{array}{r} Loss=\lambda *CELoss+\left(1-\lambda \right)*DiceLoss \end{array}$$

where *y* is the ground truth of each pixel, and *y*′ is model prediction. Dice loss function (Milletari et al., 2016) is based on dice coefficient and helps to establish the loss balance between foreground and background pixels. The loss function allocates the cross-entropy loss function and the dice loss function with λ. We set λ to 0.5 in the experiment. We use the Adam optimization (Kingma and Ba, 2014) and set initial learning rate to 0.0001. The input mini-batch size is 4. The total epoch is set to 100 with the learning rate decay strategy. Every 30 epochs, the learning rate is reduced to 1/10 of the previous value. For spatial attention module, we set the channel number of hidden feature map to 16. For channel attention module, we set the number of hidden neurons to 32.

### 4.4. Quality Measures

In order to evaluate the performance of the proposed method, we use the quality metrics commonly used in the field of medical image. Metric applies to the semantic segmentation of binary values which only considers glands as foreground, and everything else as background. Given A a set of pixels annotated as a ground truth object and B a set of pixels segmented as a gland object.

Dice Similarity Coefficient (Dice):

(10) $$\begin{array}{r} \frac{2\left(A\cap B\right)}{A+B} \end{array}$$

Jaccard Coefficient (Jaccard):

(11) $$\begin{array}{r} \frac{A\cap B}{A\cup B} \end{array}$$

Relative Volume Difference (RVD):

(12) $$\begin{array}{r} \frac{\left|B|-|A\right|}{\left|A\right|} \end{array}$$

In order to save the best model parameters during the training process, we use the Dice coefficient as the main evaluation metric. The larger the coefficient, the better the method performance. When the coefficient is 1, the predict result is consistent with the ground truth.

### 4.5. Results and Discussions

The experimental results are shown in Table 1. We compare our method with the baseline model U-Net. When our network using the channel attention (CA) alone, in the dataset GlaS, Dice score has a 0.4% improvement, and the dataset CRAG has a 0.6% improvement. When our network using the spatial attention (SA) alone, in the dataset GlaS, Dice score has a 0.9% improvement, and the dataset CRAG has a 0.6% improvement. Combining spatial and channel attention (SA+CA), there is 1% improvement on Dice score and 1.6% improvement on Jasccard score in the dataset GlaS. There is 1% improvement on Dice score and 1.4% improvement on Jasccard score in the dataset CRAG. Besides, compared with the network SegNet (Badrinarayanan et al., 2017), U-Net++ (Zhou et al., 2018), DeepLabv3+ (Chen et al., 2018), the overall performance of SCAU-Net is excellent, and it is more robust to different datasets.

**Table 1:** Our method's segmentation results compare with U-Net on dataset GlaS and CRAG.

	**GlaS**			**CRAG**		
**Method**	**Dice**	**Jaccard**	**RVD**	**Dice**	**Jaccard**	**RVD**
U-Net	0.8963	0.8175	0.0079	0.9003	0.8243	−0.0042
SCAU-Net(CA)	0.9004	0.8242	0.0190	0.9069	0.8333	−0.0072
SCAU-Net(SA)	0.9054	0.8322	−0.0166	0.9067	0.8330	−0.0033
SCAU-Net(SA+CA)	**0.9063**	**0.8332**	0.0197	**0.9100**	**0.8381**	−0.0074
DeepLabv3+	0.8866	0.7994	−0.0203	0.8672	0.7691	−0.0492
SegNet	0.7930	0.6643	−0.0582	0.8990	0.8209	−0.0030
U-Net++	0.8952	0.8166	0.0256	0.8870	0.8010	−0.0182

*CA refers to channel attention module, SA refers to spatial attention module. We also compare with the network SegNet, U-Net++, DeepLabv3+. Significant results are highlighted in bold font*.

**Figure 4:**
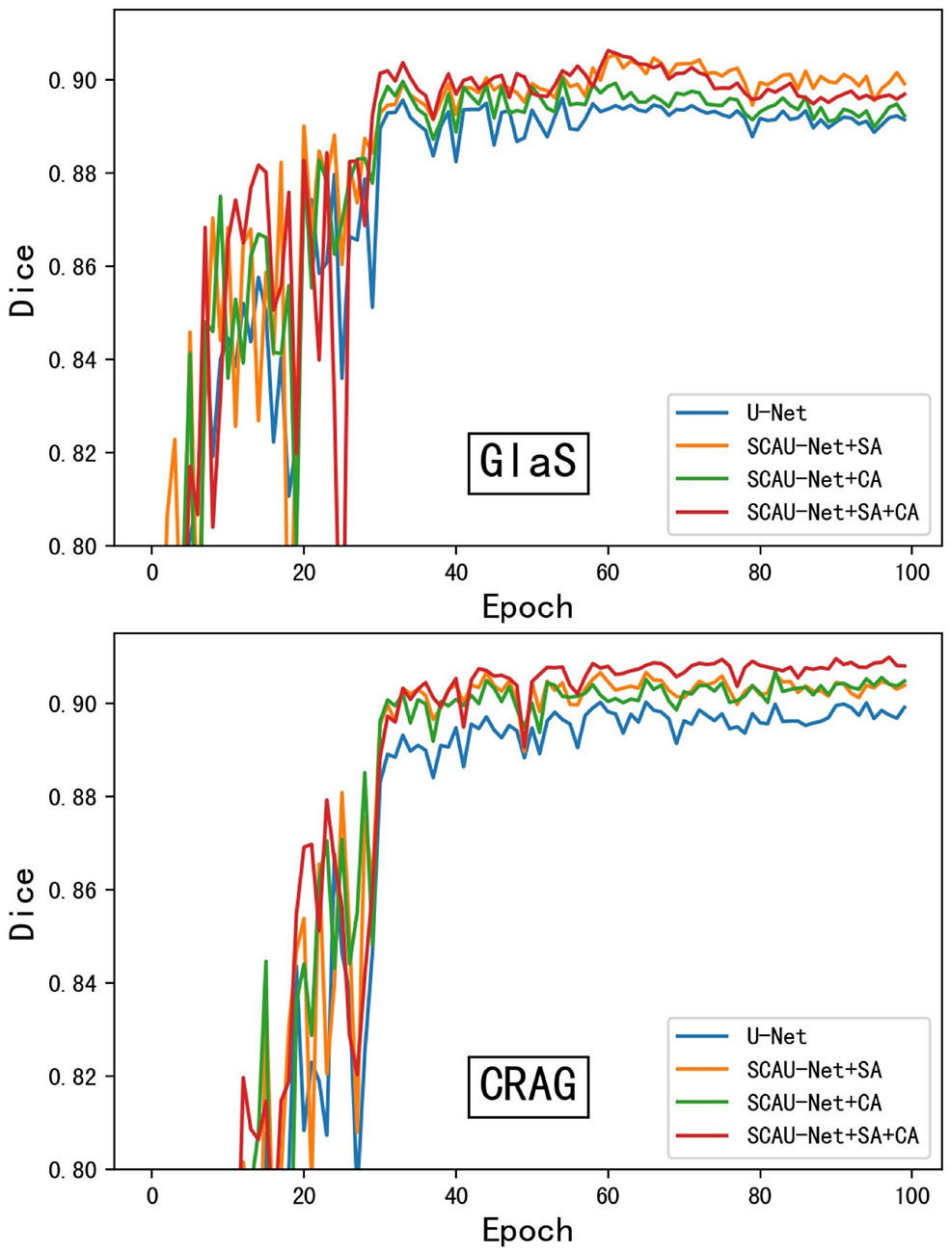
The training process of different model. Figure shows dice curve of the U-Net and SCAU-Net with different settings (CA, SA, CA+SA) on validation sets. CA, channel attention module; SA, spatial attention module.

As shown in Figure 4, we compare the training process between the U-Net and SCAU-Net. It can be observed that the SCAU-Net with spatial and channel attention (SA+CA) achieves the highest accuracy on validation sets. For the dataset GlaS, the SCAU-Net slightly over-fits after about the 60th epoch, while dataset CRAG doesn't. We analyze the results and believe that the added attention mechanism makes the model parameters increase, and the model is more likely to over-fit with less data amount.

**Figure 5:**
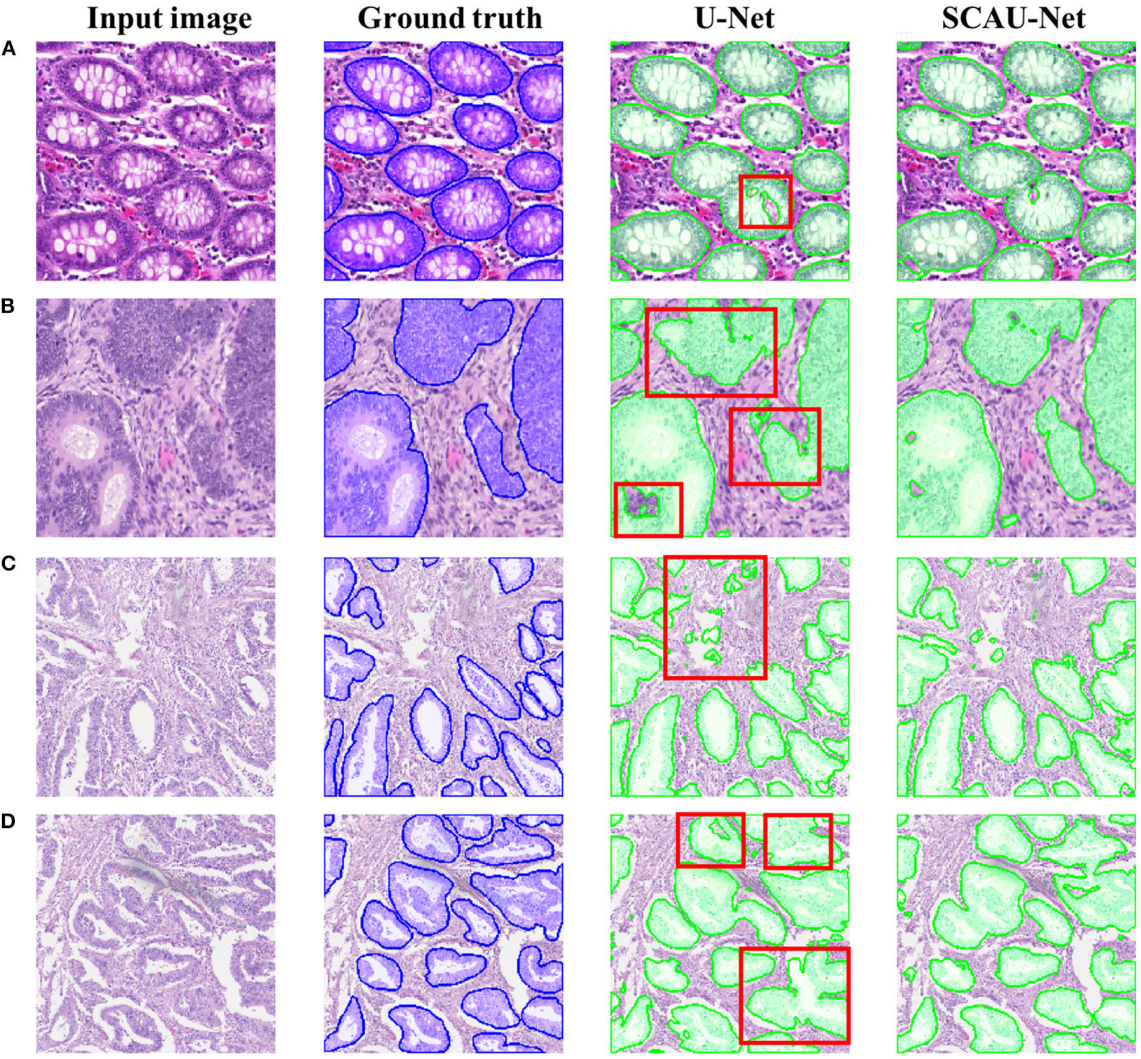
Comparison of segmentation results. Examples **(A,B)** are from the GlaS dataset, and examples **(C,D)** are from the CRAG dataset. The red boxes indicate areas with poor segmentation results.

Figure 5 shows the visualization results of the method. As shown in Figures 5A,B, for some gland objects, the U-Net network misclassifies the white area inside the gland as the background, while SCAU-Net performs better. It shows that our method has better object connectivity. For some complex scenes, SCAU-Net can accurately distinguish background noise, as shown in Figure 5C, and can distinguish the edges of multiple gland objects well to prevent “sticking,” as shown in Figure 5D. On the whole, SCAU-Net outperforms U-Net in the segmentation of glands.

**Figure 6:**
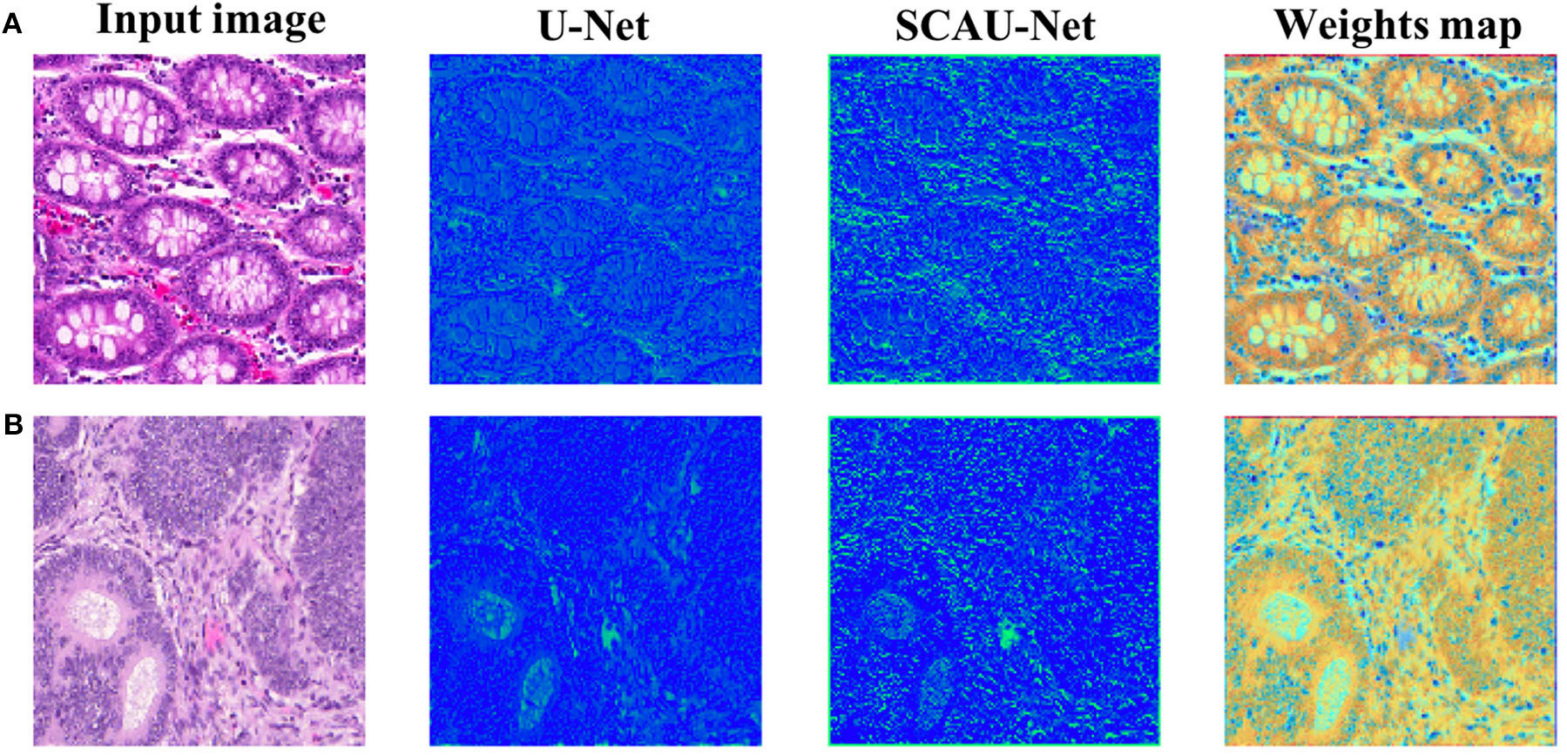
Visualization the output feature activations after Block(64). Compared with the basic U-Net network, SCAU-Net exploits spatial attention weights to recalibrate the feature map. Weights map by SCAU-Net is shown in the last column. Examples **(A,B)** are from the GlaS dataset.

In order to explore how the attention mechanism works, we visualize the effect of the model with the spatial attention mechanism added. For visual display, we extract the encoder output feature map of Block(64). Compared with the basic U-Net network, SCAU-Net exploits spatial attention weights to recalibrate the feature map. As shown in Figure 6, the feature maps extracted show the differences between the two methods. The contrast of the feature map by SCAU-Net is more prominent, indicating the wider range of values. The spatial attention weights map learned by SCAU-Net has different weight assignments in different regions, as shown in weights map. Spatial attention assigns lower weights on easily distinguishable backgrounds, non-glandular noise tissue areas, obvious contours, etc. The fuzzy boundaries of the indistinguishable contours are assigned higher weights, indicating that the network pays more attention to these difficult-to-classify regions.

## 5. Conclusion

In this paper, we extend the U-Net encoder-decoder framework, propose a new network named Spatial-Channel Attention U-Net (SCAU-Net) for image semantic segmentation. We perform the segmentation tasks on GlaS and CRAG gland dataset. The experiment results and comparisons with classic U-Net model demonstrate that our proposed model can achieve a better segmentation performance, with 1% improvement on Dice score and 1.5% improvement on Jaccard score. We also visualize the effect of attention mechanism on feature extraction to explain how the mechanism works.

In the future, the spatial and channel attention modules proposed in this paper need further exploration for the number of convolutional layers, the number of fully connected layers, and the location settings of the module embedding.

## Data Availability Statement

All datasets presented in this study are included in the article/supplementary material.

## Author Contributions

PZ proposed the main idea. JZ implemented the experiments and wrote most of the manuscript. WF and SD wrote parts of the manuscript, read, and approved the final manuscript. All authors contributed to the article and approved the submitted version.

## Conflict of Interest

The authors declare that the research was conducted in the absence of any commercial or financial relationships that could be construed as a potential conflict of interest.
